# Multiple Roles of the Stress Sensor GCN2 in Immune Cells

**DOI:** 10.3390/ijms24054285

**Published:** 2023-02-21

**Authors:** Chenxu Zhao, Han Guo, Yangxiao Hou, Tong Lei, Dong Wei, Yong Zhao

**Affiliations:** 1 State Key Laboratory of Membrane Biology, Institute of Zoology, Chinese Academy of Sciences, Beijing 100101, China; 2 University of Chinese Academy of Sciences, Beijing 100049, China; 3 Beijing Institute for Stem Cell and Regenerative Medicine, Beijing 100101, China

**Keywords:** GCN2, immune system, stress, protein kinase, tumor

## Abstract

The serine/threonine-protein kinase general control nonderepressible 2 (GCN2) is a well-known stress sensor that responds to amino acid starvation and other stresses, making it critical to the maintenance of cellular and organismal homeostasis. More than 20 years of research has revealed the molecular structure/complex, inducers/regulators, intracellular signaling pathways and bio-functions of GCN2 in various biological processes, across an organism’s lifespan, and in many diseases. Accumulated studies have demonstrated that the GCN2 kinase is also closely involved in the immune system and in various immune-related diseases, such as GCN2 acts as an important regulatory molecule to control macrophage functional polarization and CD4^+^ T cell subset differentiation. Herein, we comprehensively summarize the biological functions of GCN2 and discuss its roles in the immune system, including innate and adaptive immune cells. We also discuss the antagonism of GCN2 and mTOR pathways in immune cells. A better understanding of GCN2′s functions and signaling pathways in the immune system under physiological, stressful, and pathological situations will be beneficial to the development of potential therapies for many immune-relevant diseases.

## 1. Introduction

General control nonderepressible 2 (GCN2), which is encoded by eukaryotic translation initiation factor 2 alpha (eIF-2α) kinase 4 (EIF2AK4), is a serine/threonine-protein kinase that senses amino acid deficiencies through binding to uncharged transfer RNA (tRNA). It plays a key role in modulating amino acid metabolism in response to nutrient deprivation. Upon amino acid starvation, GCN2 promotes the phosphorylation of eIF2α, which leads to a repression of general translation and the initiation of gene reprogramming to facilitate adaptation to nutrient stress. In response to diverse stress stimuli, eukaryotic cells activate a common adaptive pathway, termed the integrated stress response (ISR), to restore cellular homeostasis [1]. ISR is a common cellular stress response that is initiated upon phosphorylation of eIF-2α at the residue serine-51 and which is critical for translational control to maintain cellular homeostasis and in response to various stress conditions in eukaryotes [2]. The four well-known sensors of ISR in mammals include GCN2, double-stranded RNA-dependent protein kinase K (PKR), heme-regulated inhibitor, and PKR-like endoplasmic reticulum kinase [3]. GCN2 activation and the phosphorylation of eIF-2α mainly inhibit the general translation of proteins not important for cell survival, promote the translation of proteins important for cell survival, and induce the transcription of several stress-responsive genes, including those involved in amino acid transport and antioxidation [4]. Recent studies have shown that GCN2 plays important roles in various physiological and pathological processes, in addition to maintaining organismal homeostasis under nutrient deprivation and other stresses. GCN2 mutations impair vascular and parenchymal remodeling during pulmonary fibrosis in rats and humans [5,6]. In this review, we summarize the recent progress on the signaling pathways and biological functions of GCN2 in mammals, with a primary focus on its diverse roles across different immune cell subpopulations.

## 2. Molecular Structure, Expression and Activation of GCN2

GCN2 was first discovered in the yeast *Saccharomyces cerevisiae* [7]. GCN2 is one of three eIF-2α kinases in yeast and one of four characterized eIF2α kinases in mammals [2,8,9]. All distinct eIF2α kinases share extensive identity in the kinase catalytic domain. In addition to the 12 conserved subdomains found in most protein kinases, they have other features including an insertion between subdomains IV and V that distinguish them from other serine/threonine kinases. Their regulatory regions share little similarity compared to the catalytic domain, which contributes to the different stress signals controlling each eIF2α kinase [10]. As an eIF-2α kinase conserved across all eukaryotes and which primarily responds to starvation of different amino acids, GCN2 is a multidomain protein. It possesses a typical eukaryotic protein kinase catalytic moiety and a paratactic domain homologous to histidyl-tRNA synthetase (HisRS), mediating the activation of GCN2 through direct interactions with multiple uncharged tRNAs that accumulate during a state of amino acid limitation [11,12]. In addition, GCN2 also contains RING finger and WD repeat containing and DEAD-like helicases (RWD) domain, partial kinase domain and C-terminal ribosome domain (CTD). RWD domain associates with the activator protein General control nonderepressible 1 (GCN1). Partial kinase domain is required for GCN2 activation. CTD is required for uncharged tRNA binding which facilitates GCN2 dimerization and ribosome association [8,13]. Recent studies have shown that GCN1 is essential for both the GCN2-dependent stress response and GCN2-independent cell cycle regulation [14]. The high concentrations of uncharged tRNAs that accumulate in starved cells favor binding to the HisRS-like domain and CTDs, which alters the conformation of GCN2, relieves inhibitory interactions between the HisRS-like domain, CTDs and the protein kinase domain and result in autophosphorylation of the activation loop of the enzyme, thereby ultimately activating GCN2 [13,15,16]. It should be noted that GCN2 isoforms lacking functional RWD domains are expressed in some organisms, such as in the parasite *T. gondii*, the malaria parasite *P. falciparum* and *Dictyostelium*, and can nevertheless be activated [17,18,19], indicating that these GCN2 isoforms are activated in a GCN1-independent way. This issue remains to be further addressed in mammals in the future.

**Figure 1:**
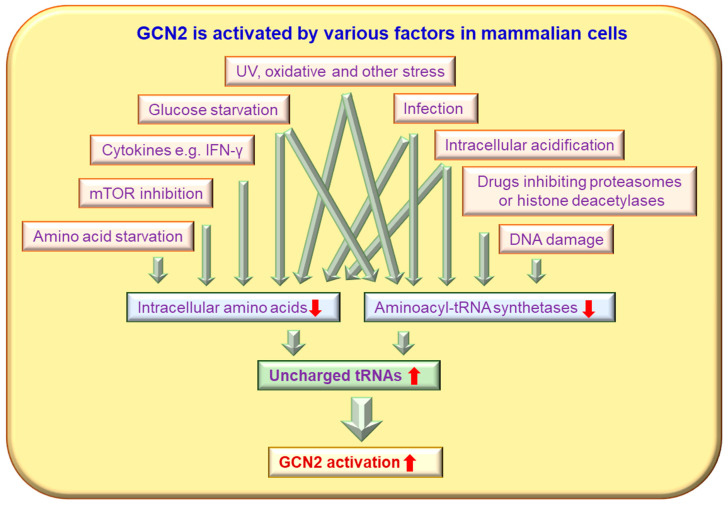
GCN2 is activated by various factors in mammalian cells. Many stimuli, such as nutrient starvation, glucose deprivation, purine starvation, high salt environment, UV irradiation, osmotic stress, high levels of urea, oxidative stress (H_2_O_2_), high salinity (NaCl), tryptophanol, anticancer drugs, and viral infections, can trigger enhanced GCN2 activity via uncharged tRNA-dependent pathways.

GCN2 expression and activation can be induced by many conditions and factors (Figure 1). The activation of GCN2 has been extensively studied, primarily in yeast, in response to amino acid starvation, including essential amino acids and some nonessential amino acids. It has recently been reported that methionine deprivation acts via a GCN2-independent mechanism, while all other essential amino acids are sensed by GCN2 in *Drosophila* [20]. Upon starvation, accumulated uncharged tRNAs bind to the HisRS-like domain of GCN2 and induce an activating conformational change [8,11,15]. Later research has demonstrated that GCN2 can also be activated by glucose deprivation, purine starvation, and a high-salt environment, as well as by stressors unrelated to nutrients, including UV irradiation, osmotic stress, high levels of urea, oxidative stress (H_2_O_2_), high salinity (NaCl), tryptophanol and other anticancer drugs that inhibit proteasomes or histone deacetylases, and viral infections [4,8,21,22,23,24,25,26] (Figure 1). Small-molecule kinase inhibitors represent a broad class of cancer therapeutics. Some Food and Drug Administration (FDA)-approved inhibitors, such erlotinib and sunitinib, can bound and activate GCN2 [27]. It has been reported that tRNA binding is required for GCN2 activation in response to UV-induced stress or oxidative stress [28]. However, ongoing translation is not required for UV or oxidative stress-induced GCN2 activation [28]. The detailed mechanisms by which these nutrient-unrelated stresses activate GCN2 need to be addressed in the future.

GCN2 can be activated by stress conditions that inhibit aminoacyl-tRNA synthetases without amino acid starvation, such as intracellular acidification, DNA damage and some antitumor drugs, ultimately leading to uncharged tRNA accumulation [29,30,31]. The inhibition of proteasome activity by MG132 or Bortezomib decreases free cysteine, asparagine, and aspartate levels in the cytoplasm, resulting in the accumulation of deacylated tRNAs and the subsequent activation of GCN2 in mammalian cells, which can be rescued by adding these amino acids to the medium. Thus, blocking proteasome function leads to GCN2 activation [32].

It is also worth noting that changes unrelated to intracellular tRNA levels can also lead to GCN2 activation. Methylglyoxal, an endogenous metabolite derived from glycolysis and harmful to cells at high concentrations, can activate GCN2 with no detectable alteration to uncharged methionyl tRNAs [33,34,35]. Rapamycin activates GCN2 without increasing uncharged methionyl tRNAs or histidyl-tRNAs [35,36,37]. Rapamycin-mediated mammalian target of rapamycin (mTOR) inhibition increases the affinity of GCN2 for amino-deacylated tRNAs [36]. The phosphorylation of eIF-2α caused by leucine starvation is dependent on maintained mechanistic target of rapamycin complex 1 (mTORC1) activity [38]. In addition, GCN2 can be activated through serine/threonine-protein phosphatase PP1-1 (Sit4) phosphatase by the treatment of cells with rapamycin, the inhibition of mTOR, or culturing cells in a poor nitrogen source, such as γ-aminobutyric acid [39]. GCN2 activity is also regulated by the phosphoinositide 3-kinase (PI3K)/protein kinase B (PKB)/glycogen synthase kinase 3 beta (GSK-β) pathway in neurons and fibroblasts [40]. Inhibition of PI3K decreases GCN2 activity, eIF-2α phosphorylation, and levels of activating transcription factor 4 (ATF4) [40]. PI3K activation inhibits GSK-3β phosphorylation, and the inhibition of GSK-3β increases GCN2 auto-phosphorylation. In turn, eIF2α phosphorylation activates the PI3K pathway indirectly [41], potentially forming a feedback loop that enables crosstalk between the PI3K pathways and general amino acid control. 

**Figure 2:**
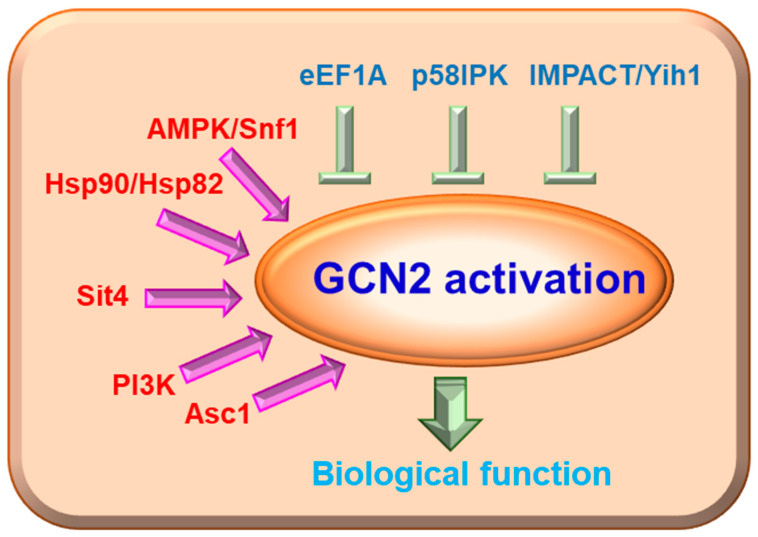
GCN2 activity is positively and negatively regulated by various molecules in mammalian cells and in yeast. AMPK/Snf1, Hsp90/Hsp82, Sit4, PI3K and Asc1 up-regulate GCN2 activity, whereas eEF1A, p58IPK and IMPACT/Yih1 inhibit GCN2 activity.

GCN2 activity in cells must be effectively inhibited under normal circumstances to allow for the maximum rate of protein synthesis. Many molecules have been recognized to directly or indirectly modulate GCN2 activation (Figure 2). Researchers have identified several endogenous inhibitors of GCN2 kinase in yeast and mammals, including eukaryotic translation elongation factor 1A (eEF1A), yeast impact RWD domain protein (IMPACT) homolog 1 (Yih1), IMPACT and p58^IPK^. eEF1A has been confirmed as an inhibitor of GCN2 in *Saccharomyces cerevisiae* cells. Visweswaraiah et al. proved that the essential translation elongation factor eEF1A can interact with the CTD of GCN2, and this interaction can be attenuated by uncharged tRNAs when cells are faced with amino acid scarcity. Meanwhile, eEF1A reduces the ability of GCN2 to phosphorylate its substrate, eIF2α, possibly through preventing GCN2 from binding eIF2α; however, it does not affect the autophosphorylation of GCN2 [42]. IMPACT and its homolog in yeast, Yih1, are potent suppressors of GCN2. Both IMPACT and Yih1 contain an RWD domain that competes with GCN2 for GCN1 binding, which is vital for GCN2 activation [43,44]. IMPACT and its homolog function mainly through the GCN2 signaling pathway. For example, IMPACT has been found to be preferentially expressed in the nervous system, where it may modulate neurite outgrowth in a GCN2-dependent way [45]; Rafael C. Ferraz et al. found that knockdown of the *IMPACT* homolog *impt-1* in *C. elegans* activates the ISR pathway and increases both lifespan and stress resistance in a GCN2-dependent manner [46]. In addition, p58^IPK^ is another inhibitor of GCN2, which can suppress GCN2 phosphorylation and prolong protein synthesis under endoplasmic reticulum deficiency, hypothermic challenge, and prolonged culture stress conditions [47].

Chaperone proteins, such as heat shock protein 82 (Hsp82) in yeast and mammalian heat shock protein 90 (Hsp90), may play an essential role in the maturation of GCN2 to an active kinase [42,48]. A highly conserved AMP-activated serine/threonine protein kinase (AMPK) in mammals and sucrose non-fermenting 1 (Snf1) in yeast, is activated when the AMP/ATP ratio increases. This process will switch off energy-consuming anabolic pathways while turning on ATP-producing pathways [49]. Snf1 and GCN2 directly interact with each other. Snf1 is activated by amino acid starvation and is required for GCN2 auto-phosphorylation and eIF-2α phosphorylation [50]. Resistance to glucose repression protein 1 (REG1) is a negative regulator of Snf1, and REG1 deletion increases the phosphorylation of GCN2 Thr-822 and eIF-2α independent of Snf1 under amino acid starvation [51]. Receptor for activated C-kinase (Rack1) in mammalian cells and its orthologue activating signal cointegrator–1 (Asc1) in *S. cerevisiae* are highly conserved proteins consisting of seven tryptophan-aspartate repeats [52] that act as scaffolds for proteins in various signaling pathways and play essential roles in regulating a wide array of biological processes. Asc1 is required for starvation-induced GCN2 auto-phosphorylation and eIF-2α phosphorylation promoting expression of amino acid biosynthesis genes in *Schizosaccharomyces pombe* under amino acid starvation [53].

## 3. GCN2 and Its Associated Signaling Pathways

**Figure 3:**
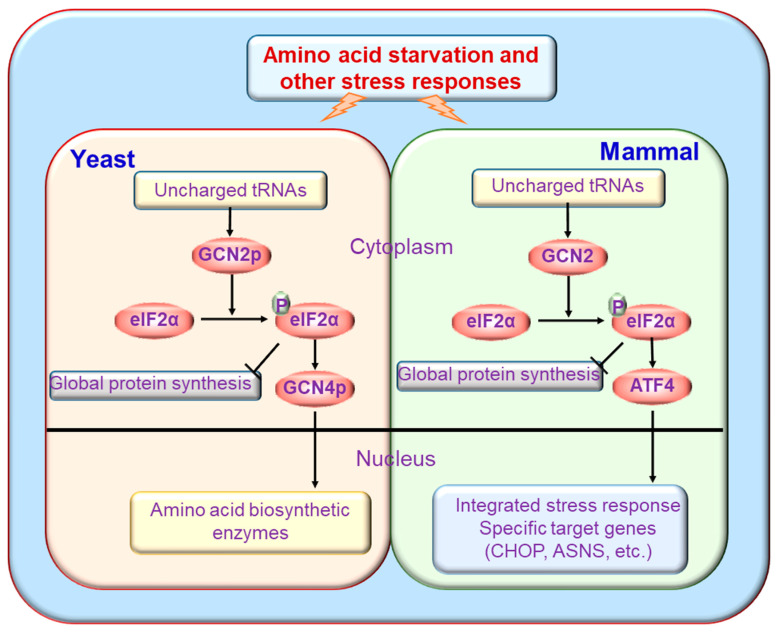
GCN2 orchestrates protein synthesis in mammalian cells and yeast. Many stimuli, such as nutrient starvation, will trigger enhanced GCN2 activity, which will subsequently inhibit global protein synthesis and promote the integrated stress response.

The eIF-2α molecule is the most important known target of GCN2 identified to date (Figure 3). The GCN2 protein can phosphorylate eIF2α in *Drosophila* and mice in vitro [10,54,55]. After being activated in response to nutritional deprivation or other stresses, GCN2 reduces eukaryotic initiation factor 2 (eIF-2) activity by phosphorylating the α subunit of eIF-2 at serine 51, which impairs the rate of GDP-GTP exchange catalyzed by the initiation factor eukaryotic translation initiation factor 2B (eIF2B) and blocks eIF-2 recycling [11,56,57]. Phosphorylated eIF-2α is unable to fulfill its normal role in helping the 40S ribosome subunit acquire methionyl initiator tRNA, and formation of the eIF2/tRNAiMet/GTP ternary complex, which is required for polysome formation and translation initiation, is decreased [58,59]. Furthermore, temperately increased phosphorylation of eIF-2α can completely inhibit protein synthesis initiation while favoring selective translation of some messenger RNA (mRNAs), including general control nondepressible 4 (GCN4) in yeast and ATF4 in mammals [60] (Figure 3).

**Figure 4:**
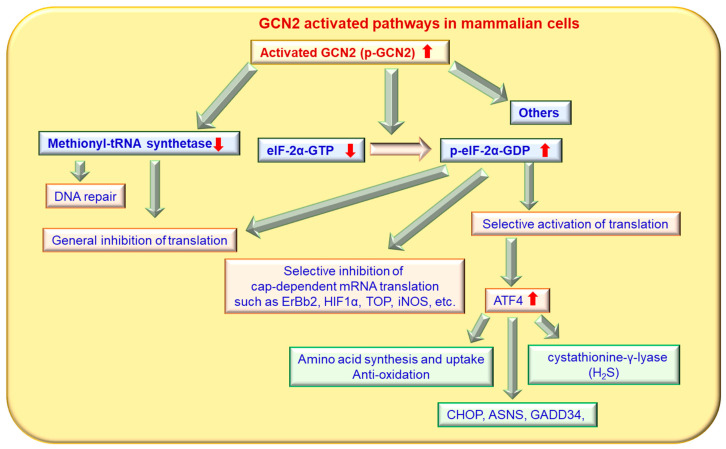
Activated GCN2 impacts many intracellular pathways in mammalian cells. Enhanced GCN2 activity can enhance methionyl-tRNA synthetase, eIF-2α and other molecules to redistribute protein translation via many pathways.

In yeast, phosphorylation of eIF-2α by phosphorylated GCN2 decreases eIF-2-GTP levels and eliminates the (ternary complex) TC’s inhibitory effects of the upstream open reading frames (uORFs) of GCN4 mRNA, thereby promoting GCN4 translation and blocking general translation [61]. GCN4 is a transcriptional activator of the general amino acid control pathway and promotes the expression of genes encoding amino acid biosynthetic enzymes to remedy nutrient deprivation [15]. Similarly, phosphorylation of eIF-2α by GCN2 in mammalian cells induces the translation of ATF4 mRNA, which be classified a transcription factor in the basic leucine zipper family, which includes Gcn4. The mechanism of ATF4 mRNA translation induced by GCN2 in mammals is similar to that of *GCN4* in yeast, which is dependent on two ORFs associated with its mRNA [62,63,64]. ATF4 is a transcription factor that binds an amino acid response element located in the promoters of specific genes and induces their transcription [65]. In the mouse liver, GCN2 deletion attenuates high fat diet-induced eIF-2α phosphorylation and the induction of ATF4-C/EBP homologous protein (CHOP) to decrease the expression of peroxisome proliferator-activated receptor gamma (PPARγ), fatty acid synthase and metallothionein [66]. In addition to eIF-2α, methionyl-tRNA synthetase has also been identified as a substrate of GCN2 (Figure 4). Methionyl-tRNA synthetase plays essential roles in initiating translation by transferring methionyl to initiator tRNA. Under UV irradiation conditions, GCN2 can phosphorylate methionyl-tRNA synthetase, which inhibits its binding to methionyl-tRNA and contributes to a down-regulation of general translation [67]. A recent study using quantitative phosphoproteomics showed that GCN2 also targets auxiliary effectors to modulate translation [68]. In addition to phosphorylation of eIF2a, GCN2 also phosphorylates the beta-subunit of the trimeric eukaryotic translation initiation factor 2 gamma (eIF2G) protein complex to promote its association with eukaryotic translation initiation factor 5 (eIF5) and, subsequently, to restrict recycling of the initiator methionyl-tRNA-bound eIF2-GDP ternary complex in amino acid-starved cells [68]. 

The protein kinase GCN2 also regulates mRNA translation in mammals in an ATF4-independent manner (Figure 4). The translation of inducible nitric oxide synthase is negatively regulated by GCN2 upon UV irradiation (22). GCN2 negatively regulates translation of the oncogene erb-b2 receptor tyrosine kinase 2 (ErBb2) and of hypoxia inducible factor 1α (HIF1α), both of which are involved in the cell cycle and cell survival [69,70]. Another direct GCN2 substrate-regulating protein is methionyl-tRNA synthetase [42]. Activated GCN2 phosphorylates methionyl-tRNA synthetase at serine 662, which inhibits methionyl-tRNA synthetase activity and activates the ataxia-telangiectasia, mutated protein kinases (ATM)/ ATM and Rad3-related protein kinases (ATR) system for DNA repair [42]. GCN2 negatively controls the translation of 5′-terminal oligopyrimidine tracts (TOP) mRNAs, which encode protein biosynthesis factors, under amino acid starvation, and this process is dependent on the stress granule-associated proteins T-cell intracellular antigen 1 (TIA-1) and TIA1 cytotoxic granule-associated RNA binding protein-like 1 (TIAR) [71]. This regulation will re-direct TOP mRNAs from polysomes to stress granules, thereby allowing modulation of limited resource usage according to amino acid availability [71]. GCN2 can directly regulate forkhead box O class protein (Foxo) activity in human cells and in *Drosophila* [72]. However, protein kinase RNA-like ER kinase (PERK) can compensate for potential deficiencies in Foxo activity when GCN2 is absent in human cells [72]. GCN2 can directly interact with Foxo3 in cells and phosphorylate Foxo3 to promote its nuclear translocation and transcriptional activation in skeletal muscle [73]. Under certain conditions, IFN-γ has the ability to promote cancer initiation and progression [74]. Importantly, IFN-γ accelerates arginine depletion and induces malignant transformation through the NF-κB/GCN2/eIF-2α pathway in vitro and in vivo [75], and arginine addition can rescue these effects of IFN-γ [76]. GCN2 activation reduces the activity of succinate dehydrogenase, an iron-sulfur mitochondrial enzyme, and promotes the nuclear localization of the transcription factor DNA-binding transcription factor 1 (Aft1) in the yeast *Saccharomyces cerevisiae* [77]. In normal situations, an excess of amino acids would promote low levels of GCN2 activity, leading to inhibition of Aft1 and iron transporters. This repression of iron transporters could help prevent intracellular iron from reaching toxic levels due to reactive oxygen species (ROS) production [77]. GCN2 is responsible for pro-apoptotic TNF-related apoptosis-inducing ligand receptor 2 (TRAIL-R2) upregulation, caspase-8 activation, and extrinsic apoptotic cell death in tumor cells under glutamine or methionine starvation [78].

In addition, the GCN2/ATF4 pathway is involved in amino acid starvation-induced expression of the stress-inducible gene p8 through an amino acid response element in the promoter of this gene [79]. Importantly, GCN2 also plays roles in regulating the amino acid starvation response in the nucleolus, in addition to its function in the cytosol. The nucleolar localization of GCN2 regulates small RNA transcripts, which may serve as alternative stress-sensing machinery for nutrient deficiency. Depletion of GCN2 by siRNA increases small RNA transcripts and induces activation of the p53 pathway, which is dependent on B-related factor 1 [80]. 

## 4. Biological Functions of GCN2

GCN2-deficient mice are viable and fertile. These mice display no obvious phenotypic abnormalities unless fed a diet lacking a single amino acid [81,82]. However, GCN2 has a wide array of biological functions across different cells and species [3,8,26,83,84]. GCN2β isoform (GCN2β), which is highly abundant in unfertilized mouse eggs, is active and elevates the phosphorylation of eIF-2α; however, phosphorylation of eIF-2α is reduced drastically after fertilization, suggesting that GCN2-mediated translational control may contribute to the regulation of oocyte maturation [85]. GCN2-mediated phosphorylation of eIF-2α is elevated in old/aging cells, potentially contributing to global translation reduction and lifespan extension [86]. GCN2 deficiency attenuates induced hepatic steatosis and insulin resistance in mice fed a high-fat diet, partially through shifting lipolysis to lipogenesis, as well as by decreasing oxidative and endoplasmic reticulum stress [66]. GCN2 is required for the growth of prostate cancer cells and regulates the expression of over 60 solute-carrier genes, including those involved in amino acid transport [87]. Inhibiting GCN2 in arginine-deprived hepatocellular carcinoma cells promotes a senescent phenotype, rendering these cells vulnerable to senolytic compound-induced death [88]. Heritable pulmonary veno-occlusive disease is related to biallelic mutations in *EIF2AK4* which encodes GCN2. GCN2 deletion impairs the ability of hematopoietic stem cells to repopulate and regenerate by regulating metabolic alterations [89]. The GCN2 activator halofuginone significantly prevents cell proliferation and inhibits expression of anti-HLA class I antibodies-induced IL-8, monocyte chemoattractive protein-1, and transforming growth factor-beta 1 in human glomerular endothelial cells, suggesting that GCN2 activation may have a protective effect against antibody-mediated graft rejection [90]. Herein, we will briefly summarize the involvement of GCN2 in oxidative stress, cell survival, autophagy, and cell metabolism.

### 4.1. GCN2 and the Oxidative Stress Response

Excessive ROS production or impaired ROS reduction efficiency can lead to oxidative stress. Oxidative stress is associated with cytotoxic effects and has been implicated in the etiology of various diseases. The oxidative stress response is highly associated with amino acid supply. GCN2 is a potential regulator of redox homeostasis. Arriazu et al. demonstrated that amino acid deprivation decreases intracellular ROS levels in hepatic stellate cells in vitro, with this effect shown to be dependent on GCN2 but not on downstream eIF-2α [91]. Deletion or pharmacological inhibition of GCN2 significantly delays collective cell migration and wound closure by impairing the maintenance of intracellular free amino acids, particularly cysteine, as well as disrupting Ras-related C3 botulinum toxin substrate 1 (RAC1)-GTP-driven ROS generation, lamellipodia formation, and focal adhesion dynamics in human epidermal keratinocytes during wound healing [92]. Furthermore, GCN2 and its downstream signaling pathway play important roles in protection against oxidative injuries induced by an amino acid imbalanced diet [93]. 

### 4.2. GCN2 and Cell Survival, Cell Cycle and Cell Differentiation

The GCN2 kinase has a broad impact on cell survival. The GCN2/eIF-2α/ATF4 signaling pathway can mediate cell survival [94]. GCN2 can sense stalled ribosomes and activate the eIF-2α/ATF4 downstream signaling pathway to promote ISR, which protects neurons against ribosome stalling-mediated cell death [95]. Cai et al. reported that the GCN2/eIF-2α/ATF3 signaling pathway is important for cells in the renal medulla during urea stress. The loss of GCN2 increases the sensitivity of cells to urea stress and promotes activated caspase-3 expression to decrease cell survival [24]. GCN2 deficiency decreases doxorubicin-induced cardiotoxicity by ameliorating apoptosis and oxidative stress through an eIF-2α/CHOP-dependent pathway [96].

The GCN2-associated signaling pathway seems required for development and lifespan extension. GCN2 deficiency impairs translational control and the expression of differentiation-associated genes, eventually inhibiting normal epidermal differentiation in organotypic skin culture [97]; these findings indicate that the GCN2/eIF-2α signaling pathway promotes the translational control and differential protein expression required for normal keratinocyte differentiation. Under conditions of amino acid restriction, activated GCN2 and its downstream transcription factor ATF4 mediate transcription of *4E-BP*, which regulates lifespan extension in *Drosophila* and is important for the normal development of certain tissues [98,99].

Xiao et al. found that the inhibited cell proliferation and increased apoptosis of breast cancer cells observed under leucine deprivation is dependent on fatty acid synthase (FASN). Leucine deprivation decreases *Fasn* expression by activating the GCN2/eIF-2α signaling pathway, which can inhibit sterol regulatory element-binding protein 1C (SREBP1C), a transcription factor that directly binds to the *Fasn* promoter and regulates *Fasn* mRNA abundance [100]. However, the specific mechanism by which the GCN2 signaling pathway regulates SREBP1C requires further investigation. GCN2 can also exert proapoptotic functions in cancer cells through posttranslational mechanisms. GCN2 protein levels critically determine the sensitivity of cancer cells to Na^+^, K^+^-ATPase ligand-induced apoptosis, both in vitro and in vivo. Na^+^,K^+^-ATPase ligand treatment triggers phosphorylation of GCN2 at threonine 899, which increases GCN2 protein expression by disrupting formation of the GCN2-beta-arrestin-NEDD4-like E3 ubiquitin protein ligase (NEDD4L) ternary complex. These increased GCN2 levels, in turn, aggravate Na^+^,K^+^-ATPase ligand-induced cancer cell apoptosis, which is largely dependent on a molecule downstream of GCN2, CHOP [101]. In eukaryotic cells, GCN2 plays an important role in regulation of the cell cycle under various stresses. DNA-damaging agents or nitrogen starvation induce cell arrest in the G1 phase, accompanied by phosphorylation of eIF-2α [102,103]. Hamanaka et al. demonstrated that PERK and GCN2 function cooperatively to regulate eIF-2α phosphorylation and cyclin D1 translation in fibroblasts after unfolded protein response (UPR) activation [104]. In addition, activation of the GCN2/eIF-2α signaling pathway can selectively up-regulate translational expression of a p21 transcript variant that contributes to cell cycle arrest at the G1/S phase and promotes cell survival [105].

### 4.3. GCN2 and Autophagy 

The GCN2 signaling pathway plays a major role in autophagy and the regulation of stress-induced autophagy gene expression. The GCN2 kinase and its downstream transcription factors, ATF4 and CHOP, are required to increase transcription of a set of genes implicated in the formation, elongation, and function of the autophagosome [106]. Fougeray et al. reported that GCN2 can mediate IFN-γ-induced autophagy in human renal epithelial cells. During this process, IFN-γ induces tryptophan metabolism, then activates the GCN2 kinase and downstream eIF2α, an activator of autophagy [107]. During the lactation cycle of the bovine mammary gland, autophagy is induced in bovine mammary epithelial cells (BMECs) as a survival mechanism to maintain cellular homeostasis. IFN-γ-induced autophagy in primary BMECs promotes dramatic primary BMEC transformation in vivo and in vitro [75]. GCN2/eIF-2α signaling pathway-mediated IFN-γ-induced autophagy in BMECs reduces milk synthesis, while arginine supplementation can attenuate IFN-γ-induced autophagy and restore milk protein and fat synthesis to some extent [108].

Autophagy and its regulatory mechanisms are involved in intestinal homeostasis and repair, supporting intestinal barrier function in response to cellular stress through tight junction regulation and protection from cell death [109]. Acute amino acid starvation-induced autophagy inhibits intestinal inflammation, a key mediator of the ISR, through a mechanism dependent on GCN2 [110].

### 4.4. GCN2, mTOR and Metabolism

GCN2 and mTORC1 are two amino acid-sensing kinases in mammalian cells, and their combined effects orchestrate cellular adaptation to amino acid levels. GCN2 kinase is typically activated by increased levels of uncharged tRNAs during amino acid scarcity. The mTORC1 kinase can be activated by amino acid sufficiency, and enhanced mTORC1 activity promotes high levels of translation to maintain cell growth and proliferation by phosphorylating two important translational regulators, the S6 kinase (S6K) and eIF4E-binding protein (4E-BP1) [111,112]. GCN2 and mTORC1 signaling pathways are both regulated by amino acids and share some common functions. 

These two amino acid-sensing systems are linked. mTORC1 is inactivated by certain nutrient deprivation conditions. GCN2 is involved in the inhibition of mTORC1; however, GCN2 alone is not sufficient to inhibit mTORC1 activity upon leucine or arginine deprivation, indicating the existence of additional mechanisms by which leucine and arginine regulate mTORC1 activity. Moreover, GCN2 inhibits mTORC1 activity through phosphorylation of eIF2α but independently of the downstream transcription factor ATF4 [113]. In contrast, Ye et al. reported that GCN2 inhibits mTORC1 activity through ATF4 [114]. Deprivation of various amino acids can activate GCN2, up-regulate ATF4 expression and promote expression of the stress response protein Sestrin2, which is required to sustain mTORC1 repression by blocking its lysosomal localization [114]. Furthermore, mTORC1 activity also seems to impact GCN2 activity. Pharmacological inhibition of mTORC1 leads to GCN2 activation and eIF-2α phosphorylation dependent on the catalytic subunit of protein phosphatase 6 (PP6C) [115]. Inhibition of mTORC1, either by rapamycin, TOR2 mutation or nitrogen deprivation, induces GCN2-dependent phosphorylation of eIF-2α [9]. In response to UV irradiation and oxidative stress, GCN2 is fully activated without any detectable change in target of rapamycin 2(TOR2) activity. However, during amino acid starvation, activation of GCN2 is dependent on maintained TOR2 activity, and GCN2 is required for timely inactivation of the mTOR pathway. Deprivation of glutamine, arginine, methionine, or lysine, but not of 16 other amino acids, has been shown to induce PKB activation in non-small cell lung cancer cells via GCN2-ATF4-regulated in development and DNA damage responses 1 (REDD1) axis-mediated mechanistic target of rapamycin complex 2 (mTORC2) activation [116]. Thus, the GCN2 and mTOR signaling pathways likely act in a coordinated manner to prevent the translational machinery from using excessive amounts of vital resources during nutrient-limited conditions. Nevertheless, the relationship between GCN2 and mTOR is complicates, and more research is needed to clearly define the regulatory interplay between GCN2 and mTOR. 

GCN2 is closely linked to various metabolism-associated processes, including fatty acid and amino acid synthesis, gluconeogenesis and the hexosamine biosynthetic pathway. In primary human CD4^+^ T cells, indolamine 2,3-dioxygenase (IDO) activates GCN2 kinase through tryptophan depletion, resulting in down-regulation of key enzymes directly and indirectly involved in fatty acid synthesis [117]. The GCN2/ATF4 pathway can up-regulate serine synthesis enzymes in response to amino acid deprivation, which synergizes with PKM2-dependent accumulation of glycolytic precursors to maintain serine synthesis [118]. Gluconeogenesis normally plays a key role in the maintenance of peripheral glucose homeostasis. GCN2-deficient mice exhibit reduced gluconeogenesis upon administration of pyruvate, a gluconeogenic substrate. Xu et al. reported that GCN2 is important for maintaining gluconeogenesis in the liver through regulating expression of CCAAT enhancer-binding protein-beta (C/EBPβ) [119].

## 5. GCN2 in the Immune System

Amino acid catabolism is closely related to innate and adaptive immunity. GCN2 plays vital roles in the immune system and in the maintenance of immune homeostasis [3]. For example, GCN2/ATF4 signaling pathway-induced 4E-BP contributes to innate immunity by biasing mRNA translation toward cap-independent mechanisms, thus enhancing AMP synthesis during infection [120]. GCN2-mediated ISR impacts immune homeostasis in the intestine. GCN2 controls intestinal inflammation by suppressing inflammasome activation in response to amino acid starvation. During amino acid starvation or intestinal inflammation, GCN2-mediated ISR is activated in intestinal antigen presenting cells (APCs) and epithelial cells. *GCN2* deficiency in CD11c^+^ APCs or intestinal epithelial cells leads to reduced autophagy and increased ROS levels, promoting inflammasome activation and interleukin (IL)-1β production and ultimately enhancing intestinal inflammation and T helper 17 cell (Th17) responses [110]. The regulatory roles of GCN2 in various immune cells will be discussed in the following sections.

### 5.1. GCN2 in Innate Cells

GCN2 participates in the protective innate immunity and in organismal stress responses caused by pathogen-induced amino acid starvation. During the process of bacterial infection, such as by *Shigella* or *Salmonella*, damage to the host cell membrane triggers an acute intracellular amino acid starvation response, then activates the GCN2/eIF-2α/ATF4/ATF3 ISR pathway and simultaneously decreases mTOR activity [121]. A systems biology approach revealed that there is a significant correlation between GCN2 gene expression in peripheral blood mononuclear cells and CD8^+^ T cell responses in humans vaccinated with the yellow fever vaccine YF-17D [122]. The increase in GCN2 gene expression in PBMCs is positively correlated with the increase of the proportion of activated CD8^+^ T cells, and the authors use experiments to confirm that after treatment of PBMCs with YF-17D, eIF2α phosphorylation is enhanced over time and Stress granules appear. It shows that in the time sequence, the expression of GCN2 in PBMC increases first, and then the activation of CD8^+^T increases [122]. Furthermore, Ravindran et al. demonstrated that YF-17D-induced GCN2 activation in dendritic cells initiates autophagy and enhances antigen presentation ability to both CD4^+^ and CD8^+^ T cells [123]. No studies addressing the direct role of GCN2 in granulocytes have been reported to date. Julia et al. provide a perspective on the signaling of pattern recognition receptors (PRRs) and cytokine receptors recruits nutrient-sensing and autophagic machinery to shape the immune response to microbes. Amino acid starvation during Shigella flexneri activates GCN2, which results in an Activating transcription factor 3 (ATF3)-dependent transcriptional signature, e.g., increased CHOP.

Ravindran et al. recently reported that mice deficient in GCN2 exhibit substantially high levels of ROS and, subsequently, of the proinflammatory mediator IL-1β in response to cellular stress. This phenomenon is likely due to a lack of autophagy in GCN2-deficient mice, whereas mice fed a reduced-amino-acid diet show significantly lower levels of oxidative stress and of inflammatory responses to cellular stress [123]. Further mechanistic insights reveal that GCN2-induced autophagy interferes with oxidative stress and inflammasome activation, thereby controlling inflammation (Figure 2). It has recently been reported that GCN2 is a key driver of the induction of anti-inflammatory macrophage functional polarization and myeloid-derived suppressor cells (MDSCs) in the tumor microenvironment, depending on ATF4 and altered oxidative metabolism and myeloid-lineage deletion of GCN2 can changes in the immune microenvironment with increased proinflammatory activation of macrophages and MDSCs and IFNγ expression in intratumoral CD8+ T cells [6]. It has been reported that both IL-6 and GCN2 are required to promote neovascularization in IFN-γ-induced IDO1-expressing asialo-GM1^high^CD11c^+^Gr-1^+^CD11b^low^ cells with high autofluorescence [124]. Treatment with a GCN2 inhibitor alleviates MDSCs-related T cell suppression and restores T cell proliferation to enhance host anti-tumor immunity in mice [125]. In response to amino acid abundance, mTOR activates host protein translation, a mechanism that coronaviruses use for their own protein synthesis and replication. In contrast, the amino acid starvation sensor GCN2 activates pathways that limit inflammation and viral replication [126].

On the other hand, GCN2 signaling in myeloid cells can also promote inflammatory reactions in certain cases. The stress response kinase GCN2 promotes macrophage inflammation both in vitro and in vivo. Amino acid deficiency enhances the sensitivity of macrophages to lipopolysaccharides, as evidenced by increased IL-6 production dependent on the GCN2-ATF4 signaling pathway. In a mouse model of septicemia, mice with a myeloid cell-specific GCN2 deficiency were shown to exhibit reduced inflammatory responses and mortality, accompanied by decreased levels of TNF-α, IL-6, and IL-12 [127]. In mice, the GCN2 kinase is a key regulator of fibrogenesis and of acute and chronic liver injury induced by carbon tetrachloride. GCN2 plays roles in hepatic fibrogenesis and in the response to acute or chronic liver injury. Upon deficiency of the essential amino acid histidine, GCN2 is activated in primary and immortalized human hepatic stellate cells, resulting in decreased mRNA and protein expression of collagen type I, which is important for fibrogenesis. GCN2-deficient mice exhibit increased susceptibility to CCl4-induced acute or chronic liver damage [128].

GCN2 can be activated by IDO-driven tryptophan consumption to mediate the biological effects of IDO1 in immune cells. In vitro studies have shown that GCN2 kinase is required for IL-6 production by IDO-expressing macrophages in response to LPS stimulation in tryptophan-free RPMI 1640 medium [127]. Deletion of GCN2 specifically in myeloid cells decreases LPS-induced mouse septic mortality and decreases LPS-induced IL-6 levels, although not TNF-α production [127]. Clearance and tolerance to autoantigens produced by apoptotic cells are important to the maintenance of homeostasis and to prevent the initiation of inflammatory autoimmunity. Increased cell death and defective clearance can lead to the initiation and development of the autoimmune disease systemic lupus erythematosus (SLE) [129]. As a type of innate scavenging cell, macrophages are important for the clearance of apoptotic cell-associated self-antigens and the maintenance of self-tolerance. The tryptophan catabolizing enzyme IDO1 limits innate and adaptive immunity to apoptotic self-antigens, and IDO inhibits the inflammatory pathology caused by systemic autoimmune diseases [130]. The protein kinase GCN2, a primary downstream effector of IDO1, is involved in apoptotic cell-driven immune suppression. Upon activation of the GCN2 signaling pathway in macrophages, IDO1 enhances production of the apoptotic cell-driven anti-inflammatory cytokine IL-10 while reducing production of the proinflammatory cytokine IL-12 [130]. IDO1 fails to promote IL-10 protein expression in GCN2-deleted macrophages due to alterations in ribosomal association with cytokine mRNA transcripts [130]. Myeloid-specific deletion of GCN2 abrogates regulatory cytokine production and promotes inflammatory T-cell responses to apoptotic cell antigens and failure of tolerance induction. Consistently, myeloid deletion of GCN2 in lupus-prone mice results in increased immune cell activation, humoral autoimmunity, renal pathology, and mortality, while activation of GCN2 was found to significantly reduce these symptoms of systemic autoimmune disease [131]. Compared to wild-type mice, GCN2-deficient mice display resistance to anemia during a number of stress conditions, including hemolysis, amino acid deficiency and hypoxia. GCN2-deleted liver macrophages exhibit defects in the ATF4- NRF2 pathway that impair erythrophagocytosis and lysosome maturation, indicating that GCN2 is an important regulator of red blood cell clearance and iron recycling in liver macrophages [132].

GCN2 deletion in macrophages blocks leucine deprivation-induced browning and lipolysis in mouse white adipose tissue [133]. Further studies have revealed that GCN2 activation in macrophages reduces monoamine oxidase A expression and increases secretion of norepinephrine from macrophages to adipocytes, enhancing white adipose tissue browning and lipolysis under conditions of leucine deprivation [133]. Injection of the beta3-adrenergic receptor agonist CL316,243 and inhibition of monoamine oxidase A were both shown to increase norepinephrine levels, enhancing browning and lipolysis in white adipose tissue under leucine deprivation conditions [133]. Therefore, GCN2 expression in macrophages is involved in the balance between white and brown adipose tissues.

### 5.2. GCN2 in T Cells

GCN2 activation is important for T cell proliferation, activation, and differentiation. It has been reported that the type of amino acid transporter can dictate T cell differentiation fate and that T cells lacking the leucine transporter solute carrier family 7 member 5 (SLC7A5) are unable to evoke a robust response to antigen exposure or differentiate into effector T cells [134]. IDO inhibits T cell proliferation through the activation of GCN2 involves many pathways, such as GCN2 down-regulating TCR-complex ζ-chain, c-Myc, aerobic glycolysis and glutaminolysis levels by reducing levels of lactate dehydrogenase A (LDH-A) and glutaminase, GCN2-mediated down-regulation of key enzymes involved in fatty acid synthesis and GCN2 activated to decreasing glucose influx, and altering key enzymes involved in metabolism to decrease aerobic glycolysis and glutaminolysis [117,135,136]. In this way, IDO could be a constraining factor for alloreactive T cell proliferation and differentiation into effector T cell subtypes [136]. However, Sonner et al. found that GCN2 deficiency in T cells did not affect immunity to B16 tumors [137]. IDO-expressing plasmacytoid DCs suppress T cell proliferation through GCN2 activation in responding T cells via tryptophan catabolism. T cell-specific GCN2 deletion has been shown to weaken IDO-mediated suppression and eliminate IDO-mediated T cell anergy in vitro [138]. Consistently, proliferation of GCN2-deficient T cells is not inhibited by IDO-expressing DCs in vivo [138]. However, it has been reported that GCN2 is not involved in the suppression of anti-tumor T cell responses via tryptophan catabolism in melanoma-bearing mice [137]. It cannot be ignored the kynenurine (KYN) pathway metabolites KYN (catalyzed by IDO1) are endogenous agonists of the aryl hydrocarbon receptor (AhR). AhR can promote the development of type 1 regulatory T cells; another major source of IL-10. IL-10 is a potent anti-inflammatory cytokine that promotes differentiation, proliferation and maintenance of Treg cells, which impair CD8^+^ T cell maturation and cytotoxicity. IL10 also promote the development and proliferation of tolerogenic DCs, tumor-associated macrophages (TAMs), and MDSCs that populate the tumor microenvironment—to support angiogenesis immune escape of cancer cells [139]. The TME is the environment around a tumor. Tumors can affect their microenvironment by releasing cell signaling molecules, promote angiogenesis around the tumor and induce immune tolerance, while immune cells in the microenvironment can affect the growth and development of cancer cells [140]. 

A well-known animal model of experimental autoimmune encephalomyelitis (EAE) is widely employed to study the mechanisms and pathogenesis of multiple sclerosis. The invasion of Th1 and Th17 cells leads to central nervous system (CNS) demyelination and lesion formation during the peak of murine EAE. Spontaneous remission of EAE may occur after the peak of the disease, during which regulatory T cells (Treg) accumulate in the inflamed CNS, suppress effector T cell responses and reduce inflammation. GCN2 participates in the remission phase of EAE by affecting a variety of T cells, including effector T cells and Treg cells. Orsini et al. reported that the peak of the disease is characterized by high IDO expression and a high frequency of plasmacytoid DCs in the CNS of wild-type C57BL/6 mice. However, GCN2-deficient mice with EAE fail to reach the remission phase and are characterized by higher levels of CNS inflammation, accompanied by the increased presence of effector T cells (Th1/Th17) and a lower frequency of Treg cells [141]. Consistently, Keil et al. reported that GCN2-deficiency in mice exacerbates chronic disease progression during the remission period of EAE, mainly due to the low frequency of Treg cells. They demonstrated that GCN2 deficiency does not impact the survival, proliferation or suppressive capacity of Treg cells; rather, it decreases the infiltration of Treg cells into the inflamed CNS by reducing their ability to respond to CCL2 gradients [142]. However, GCN2-deficiency does not impact the migration of effector T cells. A subsequent study proved that the decreased infiltration capacity of Treg cells to the CNS in GCN2-deficient mice is not due to reduced surface receptor expression. These results suggest that, under an IDO-mediated immunoregulatory environment, the GCN2 kinase may be activated to restrict effector T cell responses and promote the accumulation of Treg cells in the inflamed CNS, mediating the remission phase of EAE. On the other hand, we recently demonstrated that GCN2 controls Th9 cell differentiation through a HIF1α-dependent glycolytic pathway [143]. GCN2-deficient mice are resistant to OVA-induced allergic airway inflammation [143]. Further studies should focus on the intrinsic roles of GCN2 in the differentiation and function of different T cell subsets. 

The GCN2-mediated amino acid starvation response pathway is important for Th17 cell differentiation. Th17 cells, which mainly produce IL-17, have been recognized as a type of pro-inflammatory CD4^+^ T cell and implicated in various autoimmune and inflammatory disorders. Sundrud et al. reported that halofuginone can selectively inhibit mouse and human Th17 differentiation by activating the GCN2-mediated amino acid starvation response [144,145]. Consistently, the addition of excess amino acids rescued the inhibition of Th17 differentiation by halofuginone. This halofuginone-induced GCN2-mediated amino acid starvation response significantly reduces Th17 cell differentiation and alleviates Th17-associated EAE in vivo [144]. Halofuginone can activate the GCN2-mediated amino acid starvation response by binding glutamyl-prolyl-tRNA synthetase and inhibiting prolyl-tRNA synthetase activity; this effect can be reversed by the addition of exogenous proline or glutamyl-prolyl-tRNA synthetase [146]. Other natural product derivatives in the febrifugine family have also been shown to inhibit glutamyl-prolyl-tRNA synthetase [146]. In addition, halofuginone blocks IL-23-induced signal transducer and activator of transcription 3 (STAT3) phosphorylation and IL-23-dependent proinflammatory cytokine expression in CCR6^+^ Th17 cells via activation of the amino acid starvation response pathway [147]. Thus, targeting GCN2 is an effective approach to autoimmunity prevention, a critical rationale for the development of tools to manipulate GCN2 function for the treatment of inflammatory immune disease.

In murine models of glioblastoma, GCN2 deficiency limits CD8^+^ T cell activation and cytotoxic marker expression. Adoptive transfer of GCN2-deleted antigen-specific CD8^+^ T cells failed to control tumor burden compared to wild-type CD8^+^ T cells. This is likely because GCN2-eficient CD8^+^ T cells are sensitized to become rapidly necrotic when faced by reduced levels of PKCθ and p-PKCθ. This study demonstrates the importance of GCN2 to CD8^+^ T cell function and survival in mice [148]. GCN2-deficient CD8^+^ T cells, but not CD4^+^ T cells, exhibit defects in proliferation and trafficking in vitro and in vivo, indicating that GCN2 is required for efficient cytotoxic T cell function [149]. However, using an experimental B16 melanoma model with T cell-specific GCN2 knockout mice, Sonner et al. showed that GCN2 in T cells did not affect immunity against B16 tumors, suggesting that GCN2 is not intrinsically involved in the functional alteration of tumor-infiltrating T cells [137].

## 6. Conclusions

As a kinase that can sense amino acid scarcity and other stresses, the GCN2 kinase plays important roles in a variety of biological processes and diseases. Due to the important roles of GCN2 in many immunities, researchers continue to explore GCN2 inhibitors and activators. Several GCN2 inhibitors and activators have been approved by Food and Drug Administration (FDA), including erlotinib and sunitinib [27]. Neratinib has the ability to bind and activate GCN2 [27]. Inhibition of GCN2 activity in TAM and tumor cells in the tumor microenvironment can play an anti-tumor effect. The majority of existing studies focus on the effects of GCN2 deficiency or GCN2 activation under stress conditions, and the detailed molecular mechanisms have yet to be fully illuminated. The role of GCN2 in T cells and macrophages has been the focus of current research, but little is known about its role in neutrophils. In future studies, more attention should be paid to the antagonism of GCN2 and mTOR in different immune systems. Future research efforts should emphasize the ATF4-independent signaling pathways involved in GCN2-mediated biological regulation. Further studies should also seek to define in more detail the biological functions of GCN2 in the immune system and in immune-related diseases. 

## Data Availability

Data sharing is not applicable to this article, as no datasets were generated or analyzed in the current study.

## Abbreviations

4E-BP1	eIF4E-binding protein
Aft1	DNA-binding transcription factor 1
AhR	aryl hydrocarbon receptor
AMPK	AMP-activated serine/threonine protein kinase
APCs	Antigen presenting cells
Asc1	Activating signal cointegrator–1
ATF4	Activating transcription factor 4
ATM	Ataxia-telangiectasia, mutated protein kinases
ATR	ATM and Rad3-related protein kinases
BMECs	Bovine mammary epithelial cells
C/EBPβ	CCAAT enhancer-binding protein-beta
CHOP	C/EBP homologous protein
CNS	Central nervous system
CTD	C-terminal ribosome domain
EAE	Experimental autoimmune encephalomyelitis
eEF1A	Eukaryotic translation elongation factor 1A
eIF-2	Eukaryotic initiation factor 2
eIF2B	Eukaryotic translation initiation factor 2B
eIF2G	Eukaryotic translation initiation factor 2 gamma
eIF-2α	Eukaryotic translation initiation factor 2 alpha
eIF5	Eukaryotic translation initiation factor 5
ErBb2	Erb-b2 receptor tyrosine kinase 2
FASN	Fatty acid synthase
FDA	Food and Drug Administration
Foxo	Forkhead box O class
GCN1	General control nonderepressible 1
GCN2	General control nonderepressible 2
Gcn4p	General control nondepressible 4 protein
GSK-β	Glycogen synthase kinase 3 beta
HisRS	Histidyl-tRNA synthetase
Hsp82	Heat shock protein 82
Hsp90	Heat shock protein 90
IDO	Indolamine 2,3-dioxygenase
IMPACT	Impact RWD domain protein
ISR	Integrated stress response
MDSCs	Myeloid derived suppressor cells
mRNA	Messenger RNA
mTOR	Mammalian target of rapamycin
mTORC1	Mechanistic target of rapamycin complex 1
NEDD4L	NEDD4 like E3 ubiquitin protein ligase
NRF2	Nuclear factor erythroid 2–related factor 2
ORFs	Open reading frames
PERK	Protein kinase RNA-like ER kinase
PI3K	Phosphoinositide 3-kinase
PKB	Protein kinase B
PKR	Double-stranded RNA-dependent protein kinase
PP6C	Protein phosphatase 6
PPARγ	Peroxisome proliferator-activated receptor gamma
RAC1	Ras-related C3 botulinum toxin substrate 1
Rack1	Receptor for activated C-kinase
REDD1	Regulated in development and DNA damage responses 1
REG1	Resistance to glucose repression protein 1
ROS	Reactive oxygen species
RWD	RING finger and WD repeat containing
S6K	S6 kinase
Sit4	Serine/threonine-protein phosphatase PP1-1
SLC7A5	Solute carrier family 7 member 5
SLE	Systemic lupus erythematosus
Snf1	Sucrose non-fermenting 1
SREBP1C	Sterol regulatory element-binding protein 1C
STAT3	Signal transducer and activator of transcription 3
TIA-1	T-cell intracellular antigen 1
TIAR	TIA1 cytotoxic granule-associated RNA binding protein like 1
TME	Tumor [141] microenvironment
TOP	Translation of 5′-terminal oligopyrimidine tracts
TOR2	Target of rapamycin 2
TRAIL-R2	TNF-related apoptosis-inducing ligand receptor 2
Treg	Regulatory T cells
tRNA	Transfer RNA
UPR	Unfolded protein response
Yih1	IMPACT homolog 1
